# The impact of repeated autologous infusion of haematopoietic stem cells in patients with liver insufficiency

**DOI:** 10.1186/s13287-015-0106-1

**Published:** 2015-06-11

**Authors:** Abdel-Rahman N. Zekri, Hosny Salama, Eman Medhat, Sherief Musa, Hanan Abdel-Haleem, Ola S. Ahmed, Hanan Abdel Hafez Khedr, Mai M. Lotfy, Khaled S. Zachariah, Abeer A. Bahnassy

**Affiliations:** Molecular Virology and Immunology Unit, Cancer Biology Department, National Cancer Institute, Cairo University, Kasr Al-Aini st., Fom El-Khaleeg, Cairo, 11976 Egypt; Hepatology and Tropical Medicine Department, El-Kasr Al-Aini School of Medicine, Cairo University, Cairo, 11441 Egypt; Pathology Department, National Cancer Institute, Cairo University, Cairo, 11976 Egypt

## Abstract

**Introduction:**

The worldwide shortage of donor livers has prompted the search for alternative cell therapies. Previous data from our laboratory proved a supportive role for stem cell therapy in the treatment of end-stage liver disease patients. Therefore; this study was conducted to assess the clinical and biochemical effects of repeated stem cell infusion.

**Methods:**

Ninety patients with liver cirrhosis were randomized to receive either one session treatment (G-I) or two sessions 4 months apart (G-II) of autologous haematopoietic stem cells (HSCs) transplantation and a control group (G-III) who received regular liver treatment. G-CSF was administered to transplanted patients before infusion; HSCs were isolated from 400 cc bone marrow (BM) aspirate. CD34+/CD133+ cells were purified: 50 % of the cells were infused locally in the portal vein on the same day and the other 50 % were differentiated to MSC and infused systemically in a peripheral vein (one session treatment G-I). In G-II, the same process was repeated after 4 months from the first treatment (two session’s treatment G-II). Liver function was monitored for 12 months after stem cell therapy (SCT).

**Results:**

Statistically significant improvement was reported in the transplanted patients (G-1) as regards the mean serum albumin, bilirubin and INR levels which started to improve after 2 weeks of treatment and continued to improve till the 6^th^ month in the single infusion group. The two sessions infused group (G-II) showed sustained response which continued throughout the all follow-up period (12 month). By the end of the study, 36.7 % of the patients in G-I and 66.7 % in G-II showed improvement in the degree of ascites compared to the control group (G-III). We also reported an improvement in the hepatic functional reserve as assessed by the Child-Pugh and MELD score. Safety of the procedure was evidenced by the low incidence of complications encountered.

**Conclusion:**

In patients with end-stage liver disease, the repeated infusion with combined routes portal and peripheral veins has a beneficial effect on liver functions with minimal adverse events and more lasting clinical efficacy after repeated HSCs infusion.

## Introduction

Cirrhosis, the end result of long-term liver damage, and its related morbidity place a significant burden on health care worldwide. Liver transplantation is the only definitive therapeutic option for these patients. However, the paucity of donors, rejection and the high costs are hindering factors. Cell-based regenerative therapies, particularly the adult haematopoietic stem cell (HSC)-based therapies, are evolving as viable clinical alternatives [1].

Stem cells are clonogenic, self-renewing cells, capable of differentiating into multiple cell lineages [2]. During tissue injury, bone marrow stem cells (BMSCs) are mobilized and migrate to the injured organ. This has formed the basis for regenerative therapy whereby treatment with appropriate stem cells might ameliorate specific diseases [3].

The observation that hepatocytes could be derived from bone marrow (BM) cell populations, and further advances in the understanding of HSC plasticity, have formed the basis for stem cell therapy in patients with liver disease [4–6]. However, the mechanism by which HSCs contribute to liver repair is controversial. Initial studies suggested that adult stem cell plasticity and their differentiation to hepatocytes is a possible mechanism of action [7]. Other studies have shown that conversion to hepatocytes may occur via cell fusion [8].

Animal studies demonstrate that infusion of BM during liver injury reduces the amount of liver scarring, owing to matrix metalloproteinase-9 expression [9]. Recently, the concept of stem cell infusions exerting a paracrine proliferative effect on endogenous hepatocytes has been gaining support, backed up by rodent and human studies [10, 11].

In Egypt, results obtained from several studies suggested that autologous CD34^+^ cell transplantation offered considerable improvement in the quality of life and liver functions in patients with viral or non-viral causes of end-stage liver disease with no procedure-related complications [12]. However, it was observed that serum albumin, bilirubin and International Normalized Ratio (INR) levels had gradually worsened towards pre-infusion levels after an initial improvement for about 3–6 months, especially in patients with viral aetiologies [13].

The inability to maintain the initial improvement in those patients could be attributed, at least potentially, to the ongoing disease processes affecting the regenerated hepatocytes over a longer period. Therefore, in order to maintain benefit of such therapies, it may be important to treat the causative disease, such as viral hepatitis, to make the infused cells resistant to viral infection [14] or to repeat the injection of stem cells at intervals to achieve significant and lasting clinical results [15].

Currently, there are no published trials regarding the beneficial effect of repeated infusion of HSCs in liver disease. However, repeated infusion of BM cells in patients with large acute myocardial infarction resulted in a significant increase in left ventricular ejection fraction and a decrease in myocardial infarct size compared with single intracoronary injection [16].

## Methods

### Studied groups

This prospective study included 90 patients with hepatitis C virus (HCV)-associated liver cirrhosis who were eligible for treatment intervention. Patients were recruited from the Tropical Medicine Department, Cairo University Hospital, Cairo, Egypt during the period from May 2010 to May 2012. Written informed consent was obtained from each patient included in the study regarding the study plan or publication.

Patients were randomized into one of three groups:

1. Group 1 (G-I: one-session treatment) included 30 patients who received one-session treatment of autologous HSC transplantation, as illustrated in Fig. 1.

**Fig. 1:**
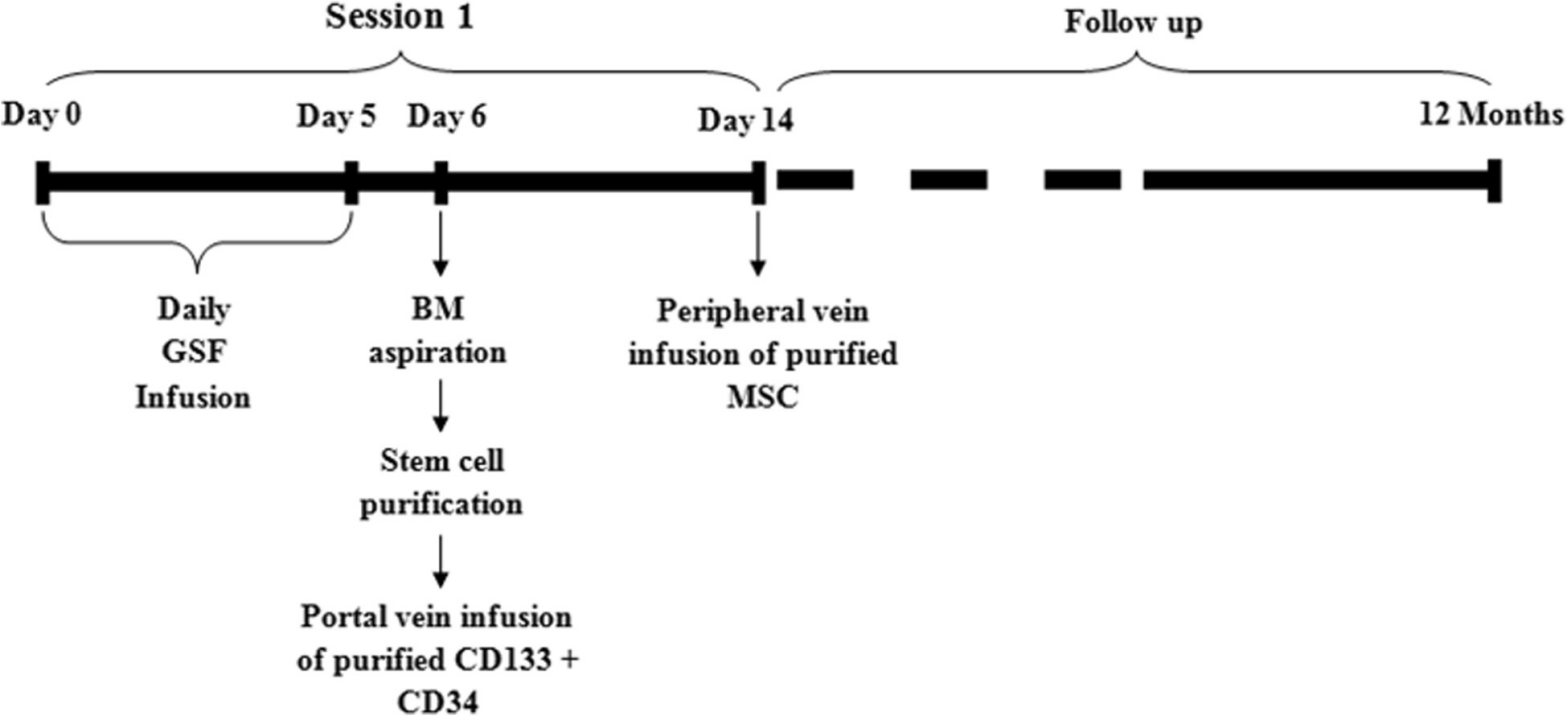
Stem cell treatment schedule of patients who received one session. *BM* bone marrow, *G-CSF*, *MSC* mesenchymal stem cell
2. Group 2 (G-II: double-session treatment) included 30 patients who received two sessions of autologous HSC transplantation 4 months apart (Fig. 2).

**Fig. 2:**
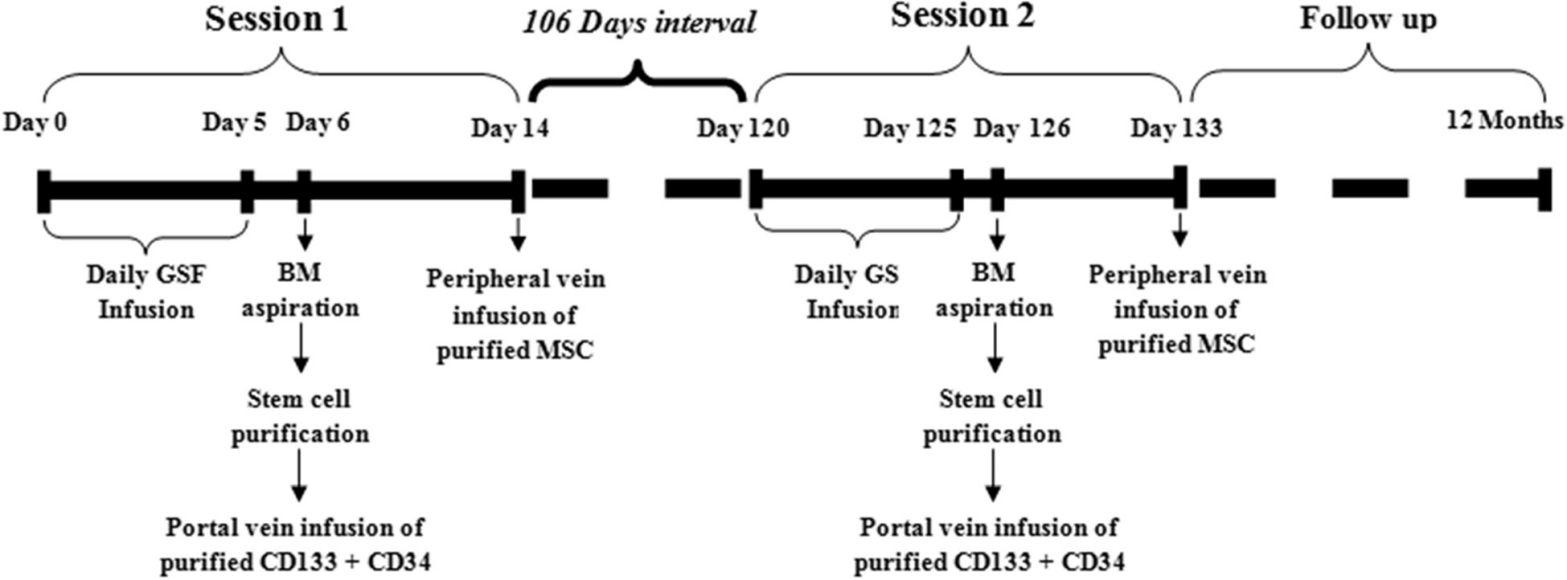
Stem cell treatment schedule of patients who received two sessions. *BM* bone marrow, *G-CSF*, *MSC* mesenchymal stem cell
3. Group 3 (G-III: control group) included 30 patients who received regular liver treatment only.

All patients in the three groups were followed for at least 48 weeks or until they died.

### Inclusion criteria

Patients included in the study fulfilled the following criteria: male or female, age range from 20 to 60 years, evidence of chronic liver insufficiency (decreased s-albumin and/or increased bilirubin and/or increased INR, Child–Pugh scores B and C and Model for End-Stage Liver Disease (MELD) scores >14) and who cannot receive a liver transplant owing to organ shortage and/or high cost of liver transplantation in Egypt. All had a World Health Organization (WHO) performance score ≤2 and were able to give written informed consent. All patients were post-HCV infection with viral load ranging from 3690 to 954,473 IU (mean = 523,764 IU). Some patients received interferon/ribavirin with no response (16 patients were previously non-responders in group I, five patients in group II and six patients in the control group), and others did not receive previous interferon therapy.

To manage HCV in the control group the participants were given virostatic drugs only (ribavirin and amantadine sulphate), and for ascites they received a diuretic combination (furosemide and spironolactone) with therapeutic paracentesis for some of them. Patients with grade III–IV varices who showed signs of impending rupture prophylactic sclerotherapy and/or band ligation underwent treatment before stem cell therapy. No hepatitis B virus cases were recruited. All patients were negative for portal tract thickening that is characteristic for schistosomiasis. No liver biopsies were performed to decrease the probability of complications since the aim of the current study is to assess the improvement in the synthetic function of the liver and not to assess the fibrotic process within the liver.

### Exclusion criteria

Patients were excluded from the study if they: were aged younger than 20 or older than 60 years; were pregnant or lactating women; had recent and/or recurrent upper gastrointestinal bleeding or spontaneous bacterial peritonitis (SBP) within 1 month before the procedure or hepatocellular carcinoma (HCC); were patients with portal vein thrombosis (PVT) on Doppler ultrasonography or severe co-morbid diseases (e.g. renal or cardiac disease); had evidence of human immunodeficiency virus or other life-threatening infection; were unable to give written consent; had a history of hypersensitivity to granulocyte colony-stimulating factor (G-CSF); or were included in any other clinical trial within the previous 6 months.

Patients in the three groups were comparable for baseline characteristics including age and sex, aetiology of liver disease and MELD score. Clinical evaluation was carried out for all patients, including a detailed medical history and complete clinical examination with special emphasis on the presence of an evidence for liver cell failure (e.g. ascites), jaundice, lower limb oedema, bleeding tendency, or signs of encephalopathy in addition to the WHO performance score.

Laboratory investigations were done for all patients including the liver biochemical profile (s-bilirubin, s-albumin, INR, alanine transaminase (ALT) and aspartate transaminase (AST)), serum creatinine levels, complete blood count (haemoglobin, white blood cell count, platelet count), coagulation profile (INR), α-fetoprotein (AFP) and hepatitis serological profile (hepatitis B surface antigen (HBsAg), hepatitis B core antibody (HBcAb), HCV-antibody and HCV-RNA by quantitative PCR and HIV by ELISA). Abdominal ultrasound scanning was performed for the three groups using a Hitachi 515 (Chiyoda, Tokyo, Japan) real-time scan after overnight fasting (before and following HSC transplantation), and the MELD score and Child–Pugh score were calculated to assess the degree of hepatic decompensation for every patient. Computed tomography (CT) scan, Doppler scan and upper gastrointestinal endoscopy were performed before stem cell transplantation.

### Ethics

The ethical committees of Kaser El-Aini School of Medicine and the National Cancer Institute, Cairo University approved the study protocol, which was in accordance with the Declaration of Helsinki and written informed consent was obtained from each patient prior to enrolment in the study. WHO Universal Trial Number: U1111-1134-8652. ClinicalTrials.gov: NCT01729221. Registered 17 November 2012, study start January 2010.

### Treatment sessions

- *G-CSF injection*. Patients from G-I and G-II received a daily subcutaneous injection of G-CSF given as filgrastim 300 μg (Neupogen® 1 ml vial; Roche Products (New Zealand) Limited PO Box 109113 Newmarket Auckland 1149 New Zealand) for 5 days to increase the number of circulating HSCs. All patients were screened at days 1, 3 and 5 by clinical assessment for adverse effects and laboratory investigations (blood count and liver biochemical profile)
- *BM stem cell aspiration*. After 5 days of G-CSF injections, a standard BM aspiration procedure was performed by a trained haemato-oncologist. The patient’s skin was cleaned with 70 % alcohol at the iliac crest, which is the usual site for puncture in adults. The skin, subcutaneous tissues and periosteum overlying the selected site for puncture were infiltrated with xylocaine local anaesthesia and serial punctures from multiple sites were performed. With a boring movement, needles (Salah and Klima New Delhi - 110055, Delhi, India) were passed perpendicularly into the cavity of the ileum at a point just posterior to the anterior superior iliac spine or 2 cm posterior and 2 cm inferior to the anterior superior iliac spine to aspirate 400 ml BM into a sterile heparin-coated container. The collected BM products were transferred to the stem cell laboratory for immuno-magnetic purification of the CD34^+^ and CD133^+^ stem cell population using the positive cell selection kit (MACS System Milteny Biotec, Gmbh, Bergisch Gladbach Germany) [17]. The isolated CD34^+^/CD133^+^ cells were washed twice with phosphate-buffered saline (pH 7.4) supplemented with 0.5 % bovine serum albumin and 5 mmol/l ethylenediamine tetraacetic acid, and centrifuged at 1500 rpm for 10 minutes at 4 °C. Cells were counted, adjusted to 1 × 10^8^, centrifuged and re-suspended in 100 ml physiological saline. On the same day, 50 % of the cells (0.5 × 10^8^) were infused locally under sonographic guide in the portal vein and the other 50 % were cultured for mesenchymal stem cell (MSC) differentiation.
- *MSC differentiation*. CD34^+^/CD133^+^ cells were cultured in DMEM/HamF12/MSC media (25 %/25 %/50 %) containing 10 % bovine serum albumin (Invitrogen Waltham, Massachusetts, USA), 1 % penicillin/streptomycin (Invitrogen Waltham, Massachusetts, USA), 1 ng/ml G-CSF, hepatocyte growth factor (5 ng/ml H1404; Sigma, Virginia, USA.) and liver extract (10 ng/ml, G7387; Sigma). Cells were incubated for 5–7 days at 37 °C in 5 % CO_2_ and examined with phase contrast microscopy for morphological changes characteristic of MSC differentiation. In the current study, a critical point for successful differentiation into MSCs was the relation between the number of cultured cells, the surface area of the tissue culture flasks used and the amount of the tissue culture media [18].
- *Characterization of cultured cells*. Cells were periodically examined with a phase contrast microscopy for morphological changes indicating trans-differentiation into MSCs. Aliquots of cultured cells were obtained for immunophenotypic characterization by flow cytometry using surface MSC markers (CD44-FITC, CD90-PerCP, CD29-FITC and CD105y versus CD45-PE). Cells were considered MSCs if they were negative for CD45-PE and positive for CD44-FITC, CD90-PerCP, CD29-FITC and CD105y (either one or all of them)
- *Injection of trans-differentiated MSCs*. On day 7 post BM aspiration, patients were admitted to hospital while fasting for intravenous infusion with approximately 1 × 10^6^/kg body weight of the expanded MSCs:
  (A) Treatment protocol for G-I: one-session based treatment as described above.
  (B) Treatment protocol for G-II: two-session based treatment as described above with a 4-month interval between the two sessions. Both sessions have similar procedure and dose of infused cells.
  (C) Treatment protocol for G-III (regular liver treatment): patients received their usual, regular supportive liver treatment.
- *Follow-up of patients after MSC infusion*. All patients in the three groups were followed up every hour for 24 hours, then weekly for the first month and monthly for 12 months. During the follow-up the patients were observed for clinical improvement through assessing the degree of fluid retention (ascites and lower limb edema), performance status and score. Some patients recorded a few complications such as mild bony aches and low-grade fever after receiving G-CSF which subsided spontaneously. Biochemical assessment included s-bilirubin and albumin, prothrombin time and concentration, INR, ALT and AST, s-creatinine levels and complete blood analysis, together with assessment of Child–Pugh score progression.

### Statistical analysis

All patients’ data were tabulated and processed by SPSS (Statistical Package for Science and Society) version 12.0 for Windows XP (Seattle, Washington, US). Descriptive statistics were presented as mean ± standard deviation for quantitative variables, whereas qualitative data were expressed as frequency (number) and percent. Comparisons between groups were carried out using the chi-square test, Fischer’s exact test or McNemar test when appropriate for qualitative data. Independent-sample *t* test and paired-sample *t* test were used for normally distributed quantitative variables. The non-parametric Mann–Whitney test and the Wilcoxon singed-rank test were used for abnormally distributed quantitative variables. Percent changes in prognostic variables were calculated and compared using the Mann–Whitney test. *P* <0.05 was considered statistically significant. Linear regression analysis was employed to determine the degree of improvement of the clinical features in relation to response to treatment.

## Results

The baseline clinical, ultrasonographic and endoscopic features of all studied groups are illustrated in Tables 1, 2 and 3.

**Table 1:** Baseline laboratory and clinical investigations of the studied groups

	Control group	Single infusion	Repeated infusion	*P* value
Age (years)	49.43 ± 4.53	49.63 ± 4.58	50.97 ± 4.15	1.00
Male	26 (86.7 %)	25 (83.3 %)	26 (86.7 %)	0.923
Female	4 (13.3 %)	5 (16.7 %)	4 (13.3 %)	0983
Haemoglobin (g/dl)	10.33 ± 1.06	10.2 ± 1.48	10.3 ± 0.77	0.614
TLC (1000/cm)	6.07 ± 1.64	5.17 ± 2.05	6.07 ± 1.14	0.520
Platelets (1000/cm)	104 ± 34	104 ± 55	104 ± 37	0.542
Total bilirubin (N: 0.1–1 mg/dl)	3.19 ± 0.89	3.59 ± 0.79	3.16 ± 0.99	0.326
Serum albumin (N: 3.4–5.2 g/dl)	2.8 ± 0.15	2.57 ± 0.15	2.83 ± 0.16	0.291
INR (N: 1)	1.67 ± 0.16	1.76 ± 0.22	1.68 ± 0.17	0.504
ALT folds (N: 0–41 IU)	33.3 ± 16.75	32.9 ± 15.07	33.3 ± 18.82	0.975
AST folds (N: 0–37 IU)	40.63 ± 25.37	41 ± 20.31	40 ± 29.79	0.401
AFP	5.4 ± 3.21	8 ± 3.79	5.4 ± 2.63	0.816
Serum creatinine (0.7–1.2 mg/dl)	1.05 ± 0.15	0.92 ± 0.23	1.04 ± 0.30	0.841
Jaundice	18 (60 %)	22 (73.3 %)	21 (70 %)	0.26
Encephalopathy	14 (46.7 %)	12 (40 %)	13 (43.3 %)	0.81
Lower limb oedema	20 (67.7 %)	20 (67.7 %)	19 (63.3 %)	0.45
Bleeding tendency	23 (76.7 %)	24 (80 %)	22 (73.3 %)	0.56
Haematemesis	14 (46.7 %)	13 (43.3 %)	15 (50 %)	0.82

Data presented as number (percent) or mean ± standard deviation. None of these parameters showed significant differences among the three groups

*AFP* α-fetoprotein, *ALT* alanine aminotransferase, *AST* aspartate aminotransferase, *INR* International Normalized Ratio, *TLC* total leucocyte count

**Table 2:** Ultrasonographic features of the studied groups

	Control group		Single infusion		Repeated infusion		*P* value
	*N*	%	*N*	%	*N*	%	
Liver size							
Average	6	20	6	20	8	26.7	1.00
Shrunken	24	80	24	80	22	73.3	
Liver texture							
Cirrhotic	30	100	30	100	30	100	
Spleen							
Average sized	0	0	0	0	0	0	0.427
Mild splenomegaly	12	40	18	60	24	80	
Moderate splenomegaly	18	60	12	40	6	20	
Ascites							
Absent	13	43.3	9	30	12	40	0.274
Mild	5	16.7	6	20	6	20	
Moderate	8	26.7	10	33.3	8	26.7	
Massive	4	13.3	5	16.7	4	13.3	
Portal vein							
Patent	30	100	30	100	30	100	

None of these parameters showed significant differences among the three groups

**Table 3:** Endoscopic features of the studied groups

Endoscopic findings	Control group		Single infusion		*P* value	Repeated infusion		*P* value
	*N*	%	*N*	%		*N*	%	
Varices								
None	3	10	5	16.7	0.29	4	13.3	0.8
Grade I	14	46.7	6	20	0.091	14	46.7	0.4
Grade II	6	20	9	30	0.4	6	20	0.2
Grade III	6	20	6	20	0.25	3	10	0.31
Grade IV	1	3.3	4	13.3	0.37	3	10	0.2
Congestive gastropathy	24	80	23	76.7	0.28	22	73.3	0.3

*P* >0.05 is not statistically significant, between the control and study group

**Fig. 3:**
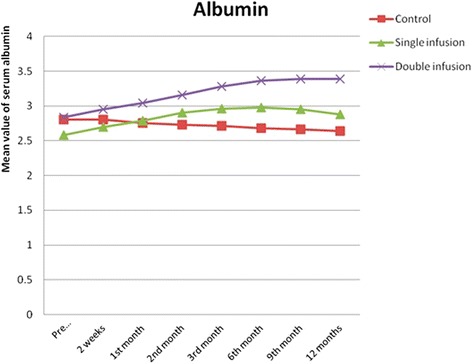
Changes in serum albumin in the studied groups before infusion, after 2 weeks and 1, 2, 3, 6, 9 and 12 months

Both single and double shot-treated patients showed increased s-albumin 2 weeks after infusion, with consistent increase after 1, 2, 3 and 4 months. As for the single infusion group, s-albumin started to decline after 12 months. The double infused group kept the s-albumin level and almost reached the normal value (3.39 ± 0.21). The difference between the two groups was statistically significant (*P* = 0.00) (Fig. 3 and Table 4).

**Table 4:** Biochemical changes in the studied groups

	Pre treatment	2 weeks	1 month	2 months	3 months	6 months	9 months	12 months
Albumin								
Control	2.80 ± 0.15	2.80 ± 0.14	2.75 ± 0.19	2.73 ± 0.18	2.71 ± 0.21	2.68 ± 0.22	2.66 ± 0.22	2.64 ± 0.19
Single infusion	2.58 ± 0.15	2.70 ± 0.22	2.79 ± 0.22	2.90 ± 0.24*	2.96 ± 0.35*	2.98 ± 0.33*	2.95 ± 0.33*	2.88 ± 0.30*
Double infusion	2.84 ± 0.17	2.95 ± 0.13^#^	3.04 ± 0.14^#^	3.16 ± 0.14^#^*	3.28 ± 0.17^#^*	3.36 ± 0.20^#^*	3.39 ± 0.20^#^*	3.39 ± 0.21^#^*
s-Bilirubin								
Control	3.19 ± 0.89	3.23 ± 0.87	3.31 ± 0.92	3.32 ± 0.92	3.33 ± 0.92	3.36 ± 0.91	3.30 ± 0.88	3.24 ± 0.78
Single infusion	3.59 ± 0.79	3.43 ± 0.77	3.39 ± 0.76	3.29 ± 0.83	3.18 ± 0.88	3.10 ± 0.93*	3.16 ± 0.81	3.18 ± 0.84^#^
Double infusion	3.16 ± 0.99	3.07 ± 0.95	3.03 ± 0.96	2.96 ± 0.94	2.86 ± 0.98*	2.76 ± 0.98^#^*	2.77 ± 1.00^#^*	2.75 ± 0.98^#^*
INR								
Control	1.67 ± 0.16	1.62 ± 0.19	1.61 ± 0.21	1.67 ± 0.19	1.73 ± 0.19	1.73 ± 0.21	1.74 ± 0.20	1.72 ± 0.20
Single infusion	1.76 ± 0.22	1.60 ± 0.32	1.54 ± 0.33	1.46 ± 0.34*	1.42 ± 0.36*	1.44 ± 0.34*	1.46 ± 0.29*	1.49 ± 0.28*
Double infusion	1.68 ± 0.17	1.60 ± 0.20	1.57 ± 0.22	1.53 ± 0.22	1.49 ± 0.16*	1.44 ± 0.17*	1.43 ± 0.18*	1.41 ± 0.15*
Serum creatinine								
Control	1.06 ± 0.15		0.95 ± 0.10	0.94 ± 0.06	0.95 ± 0.09	0.96 ± 0.14	0.95 ± 0.11	0.93 ± 0.99
Single infusion	0.92 ± 0.13		0.90 ± 0.15	0.92 ± 0.17	0.88 ± 0.16	0.89 ± 0.16	0.91 ± 0.12	0.89 ± 0.11
Repeated infusion	1.04 ± 0.16		0.93 ± 0.10	0.91 ± 0.11	0.91 ± 0.09	0.90 ± 0.13	0.91 ± 0.22	0.92 ± 0.13

Data presented as mean ± standard deviation

*P <0.05 between single infusion or repeated infusion groups and the control group

^#^ *P* <0.05 between the single infusion and repeated infusion groups

**Fig. 4:**
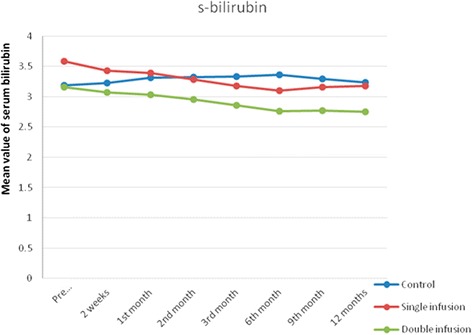
Changes in serum bilirubin in the studied groups before infusion, after 2 weeks and 1, 2, 3, 6, 9 and 12 months

**Fig. 5:**
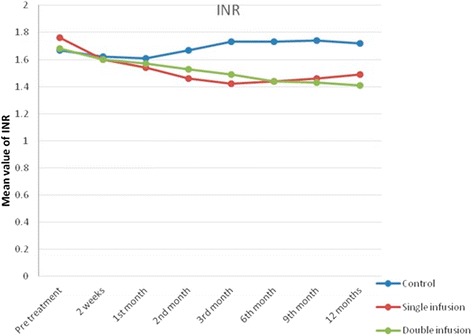
Changes in International Normalized Ratio (INR) in the studied groups before infusion, after 2 weeks and 1, 2, 3, 6, 9 and 12 months

Similarly, there was a statistically significant improvement in bilirubin level and INR in the double-infused patients compared with the single-infused patients and control group after 12 months of follow-up (Table 4 and Figs. 4 and 5). However, no statistically significant difference was found between both studied groups regarding the renal functions at baseline and all through the study.

**Fig. 6:**
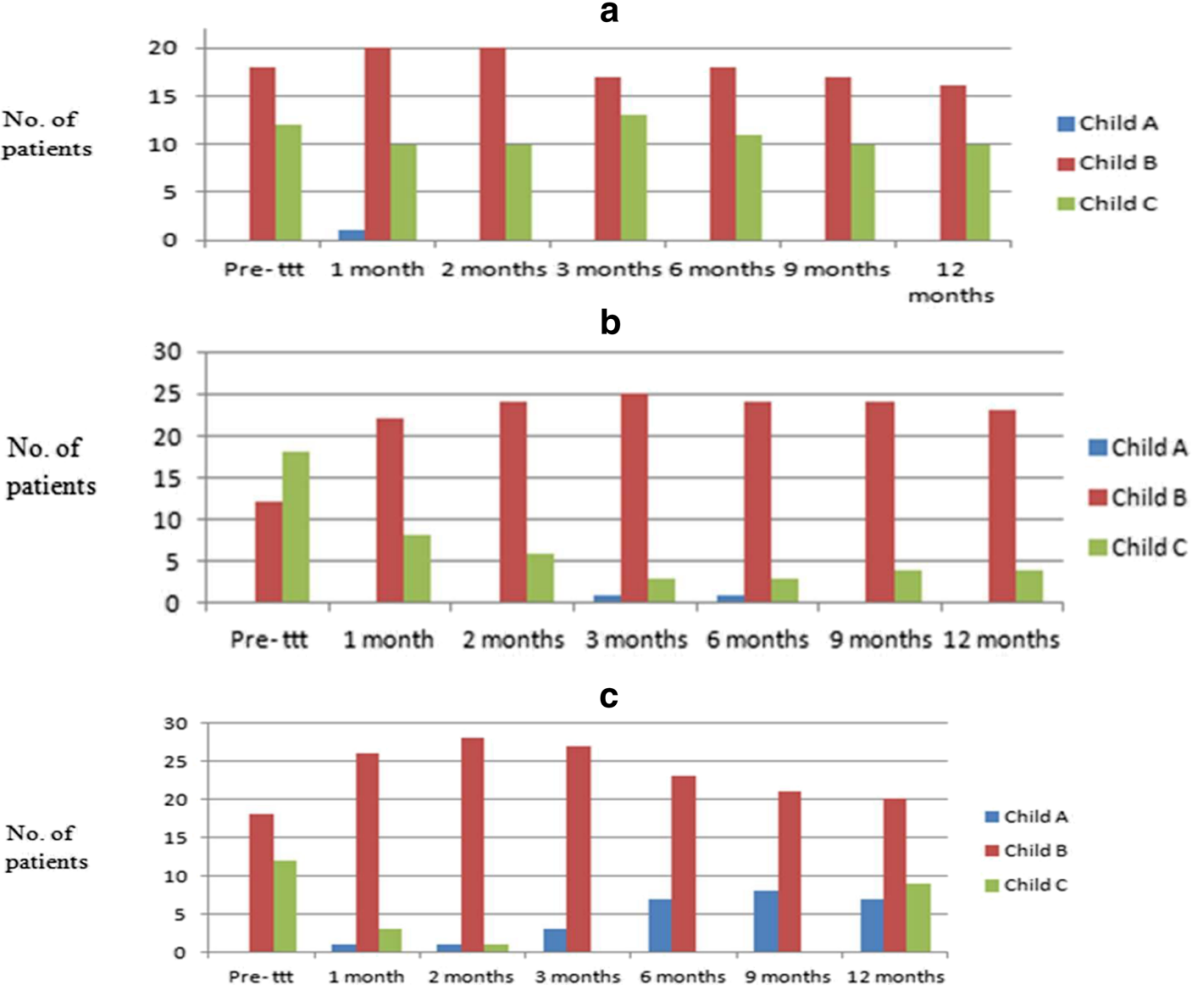
**a** Child–Pugh score in group III patients (control group). **b** Child–Pugh score in group I patients (single infusion). **c** Child–Pugh score in group II patients (repeated infusion)

**Fig. 7:**
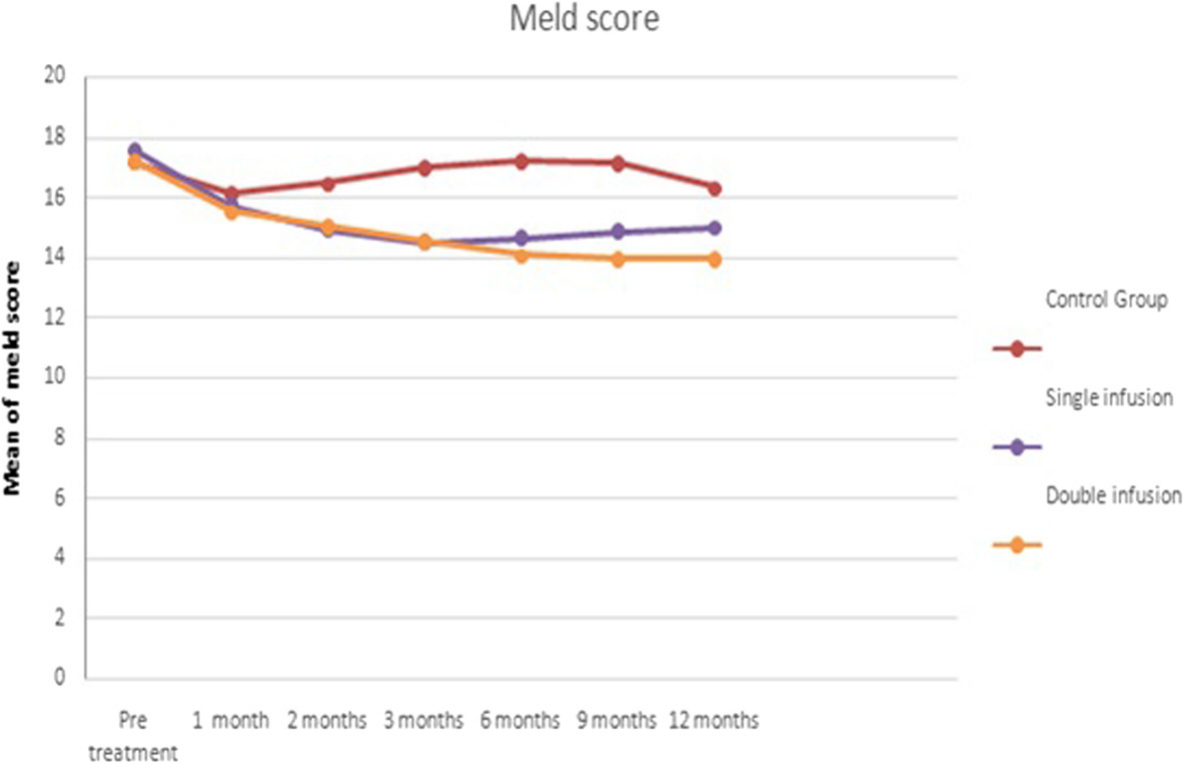
Changes in Model for End-Stage Liver Disease (MELD) score in the studied groups before infusion, after 2 weeks and 1, 2, 3, 6, 9 and 12 months

At the end of the study, 12 patients (40 %) showed improvement in their Child–Pugh grade compared with the baseline (*P* = 0.05) (Table 5 and Fig. 6). Similarly, there was an improvement in the MELD score of the double-infused group (17.23 ± 1.33) rather than both the control and the single-infused groups at the beginning of the study compared with that at 12 months in the double-infused group (13.96 ± 1.59) compared with the single-infused group (14.96 ± 2.39) and the control group (16.32 ± 3.70) (*P* = 0.0001) (Table 6 and Fig. 7).

**Table 5:** Progress of Child–Pugh score in the studied groups after 1, 2, 3, 6, 9 and 12 months of infusion

	Child–Pugh A		Child–Pugh B		Child–Pugh C		χ^2^	*P* value
	Single infusion	Double infusion	Single infusion	Double infusion	Single infusion	Double infusion		
Pre-treatment	0 (0)	0 (0)	12 (40)	18 (60)	18 (60)	12 (40)	9.73	0.259
1 month	0 (0)	1 (3.3)	22 (73.3)	26 (86.7)	8 (26.7)	3 (10)	4.72	0.03
2 months	0 (0)	1 (3.3)	24 (80)	28 (93.3)	6 (20)	1 (3.3)	4.59	0.03
3 months	1 (3.3)	3 (10)	25 (83.3)	27 (90)	3 (10)	0 (0)	1.03	0.03
6 months	1 (3.3)	7 (23.3)	24 (80)	23 (76.7)	3 (10)	0 (0)	1.43	0.03
9 months	0 (0)	8 (26.7)	24 (80)	21 (70)	4 (13.3)	0 (0)	1.54	0.04
12 months	0 (0)	7 (23.3)	23 (76.7)	20 (67)	4 (13.3)	1 (3.3)	3.37	0.05*

Data presented as count (%)

*There is a statistically significant difference between the repeated-infusion and the single-infusion groups (*P* ≤0.05)

**Table 6:** Changes in Model for End-Stage Liver Disease score in the studied groups before infusion, after 2 weeks and 1, 2, 3, 6, 9 and 12 months

Study group	Pre-treatment	1 month	2 months	3 months	6 months	9 months	12 months
Control group	17.20 ± 1.32	16.13 ± 1.54	16.47 ± 1.43	17.00 ± 1.68	17.24 ± 1.90	17.15 ± 1.95	16.32 ± 3.70
Single infusion	17.60 ± 1.90	15.73 ± 2.89	14.90 ± 3.24*	14.48 ± 3.11*	14.64 ± 2.92*	14.86 ± 2.57*	14.96 ± 2.39*
Double infusion	17.23 ± 1.33	15.53 ± 1.99	15.03 ± 1.81*	14.57 ± 1.56*	14.07 ± 1.72*	13.97 ± 1.74*^#^	13.96 ± 1.59*^#^

Data presented as mean ± standard deviation

* *P* <0.05 between single infusion or repeated infusion and control groups

# *P* <0.05 between single-infusion and repeated-infusion groups

**Fig. 8:**
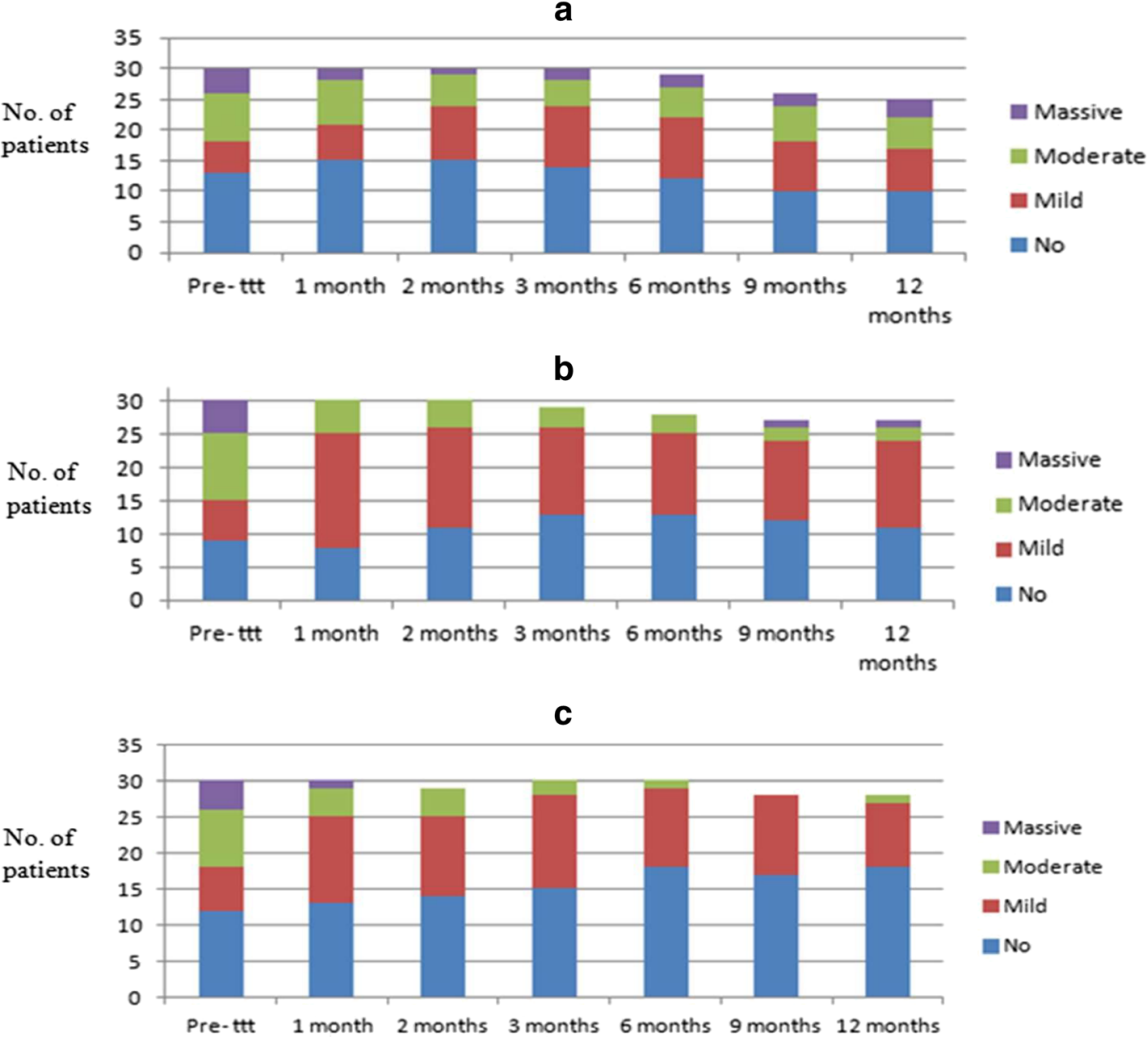
**a** Progress of degree of ascites in group III (control group). **b** Progress of the degree of ascites in group I (single infusion). **c** Progress of degree of ascites in group II (repeated infusion)

At the end of the study, 66.7 % of the patients in G-II showed improvement in their ascites compared with 36.7 % in G-I (*P* = 0.001) (Table 7 and Fig. 8). Changes in the grade of ascites were confirmed by measuring the abdominal girth and the body weight of the patients as well as by repeated ultrasonography during the follow-up. Ascites was categorized as: (1) mild if localized in the pelvis and/or hepatorenal angle only, (2) moderate if it reaches the mid-abdomen or (3) massive if more than this. On the other hand, there was a significant reduction in the tapping of ascites in G-II patients after 9 and 12 months; respectively compared with 15 % in G-I and 32 % in the control group (*P* = 0.58).

**Table 7:** Change in the degree of ascites after 3, 6, 9 and 12 months

		Control group		Single infusion		*P* value	Repeated infusion		*P* value
		*N*	%	*N*	%		*N*	%	
After 1 month	Improved	1	3.3	11	36.7	00.02	26	86.7	0.003
	Same grade	29	96.7	19	63.3	0.32	4	13.3	0.45
	Worsened	0	0	0	0	–	0	0	-
After 2 months	Improved	0	0	15	50	0.06	20	66.7	0.001
	Same grade	28	93.3	15	50	0.31	9	30	0.53
	Worsened	2	6.7	0	0	–	1	0	0.92
After 3 months	Improved	2	6.7	15	50	0.03	18	60	0.03
	Same grade	27	90	14	46.	0.62	10	33.3	0.61
	Worsened	1	3.3	0	0	–	2	6.7	0.71
After 6 months	Improved	2	6.7	14	46.7	0.002	20	66.7	0.002
	Same grade	25	83.3	14	46.7	0.35	10	33.3	0.92
	Worsened	2	6.7	0	0	–	0	0	0.92
After 9 months	Improved	0	0	12	40	0.006	20	66.7	0.001
	Same grade	26	86.7	16	53.3	0.3	10	33.3	0.46
	Worsened	2	6.7	0	0	–	0	0	0.83
After 12 months	Improved	1	3.3	11	36.7	0.003	20	66.7	0.001
	Same grade	23	76.7	16	53.3	0.42	8	26.7	0.46
	Worsened	1	3.3	0	0	–	0	0	0.92

The correction in portal hypertension, which occurred as a consequence of the anti-fibrotic effect of the infused MSCs, was not rapid. This effect together with the improvement in albumin level can explain the good response in ascites, but for portal hypertension and variceal size to change this will probably need a longer time of follow-up and may require repeated MSC infusion at different time intervals.

During the whole follow-up period there was no statistically significant difference between both studied groups regarding hepatic encephalopathy in spite of the remarkable improvement in the synthetic liver function as shown in Table 8. This is explainable since stem cells have no effect on the porto-systemic collaterals that shift ammonia away from the liver to the brain.

**Table 8:** Progress of hepatic encephalopathy in the studied groups at baseline and after 3, 6, 9 and 12 months of follow-up

Timing of hepatic encephalopathy	Number of episodes	Single infusion (Group I)		Repeated infusion (Group II)		Control group (Group III)		*P* value
At baseline	No	18	60 %	17	56.7 %	16	53.3 %	0.9
	Once	5	16.7 %	8	26.7 %	9	30 %	
	Repeated	7	23.3 %	5	16.7 %	5	16.7 %	
	Total	30	100 %	30	100 %	30	100 %	
After 3 months	No	28	93.3 %	29	96.7 %	26	86.7 %	0.13
	Once	1	3.3 %	1	3.3 %	4	13.3 %	
	Repeated	0	0 %	0	0 %	0	0 %	
	Mortality	1	3.3 %	0	0 %	0	0 %	
	Total	30	100 %	30	100 %	30	100 %	
After 6 months	No	25	83.3 %	26	86.7 %	23	76.7 %	
	Once	3	10 %	4	13.3 %	6	20 %	
	Repeated	0	0 %	0	0 %	0	0 %	
	Mortality	2	6.7 %	0	0 %	1	3.3 %	
	Total	30	100 %	30	100 %	30	100 %	
After 9 months	No	27	90 %	27	90 %	23	76.7 %	0.37
	Once	1	3.3 %	1	3.3 %	5	16.7 %	
	Repeated	0	0 %	2	6.7 %	0	0 %	
	Mortality	2	6.7 %	0	0 %	2	6.7 %	
	Total	30	100 %	30	100 %	30	100 %	
After 12 months	No	26	86.7 %	25	83.3 %	24	80 %	0.47
	Once	1	3.3 %	2	6.7 %	1	3.3 %	
	Twice	0	0 %	1	3.3 %	0	0 %	
	Mortality	3	10 %	2	6.7 %	5	16.7 %	
	Total	30	100 %	30	100 %	30	100 %	

### Survival analysis

The mortality rate in G-I (single infusion group) was 10 % (three patients, two of them died of hepatorenal syndrome (HRS) type 1 and one patient died due to uncontrolled attack of haematemesis). In G-II (repeated infusion), the mortality rate was 6.7 % (two patients): one died of HRS type I and the other from an uncontrolled attack of haematemesis. In the control group, the mortality rate was 16.5 % (five patients): two from HRS type 1, two from septic shock (following an episode of SBP) and one from an uncontrolled attack of haematemesis as shown in Table 8.

## Discussion

Pegylated interferon alpha and ribavirin are effective against HCV in the treatment-naïve patients, with sustained viral response of about 60 % in genotype 4 with undesirable adverse effects. However, these regimens are not eligible for many patients, and they are associated with poor response and adverse events in cirrhotic patients. Pegylated interferon was not used in the current study, since most of our cases were Child–Pugh score C where interferon has a limited effect with many side effects. On the other hand, the second generation (sofosbuvier, semiprivir) is not yet available in Egypt. Patients were therefore maintained on suppressive therapy using ribavirin and amantadine.

Liver cirrhosis represents the end result of chronic liver disease and is a major cause of mortality worldwide [19]. In Egypt, HCV-associated liver disease is a national health problem causing considerable morbidity and mortality [20].

Liver transplantation is the only curative treatment for decompensated cirrhosis, but it is limited by technical difficulties, high cost and lack of donors [21]. Other treatment modalities for decompensated cirrhosis are only symptomatic with transient improvement of the quality of life [22]. Consequently, the majority of untransplanted patients die while waiting for transplantation. Therefore, it is necessary to develop new therapeutic modalities for patients with chronic liver disease.

Cell-based therapy, including stem cell therapy, offers considerable hope for these patients since the stem cells, including BM-derived stem cells, have the capacity for self-renewal and multi-lineage differentiation [23]. Transplantation of BMSCs can therefore restore liver mass and function, alleviate fibrosis and correct inherited diseases [24]. In addition, patients receiving autologous BMSCs demonstrated considerable improvement in their laboratory data and quality of life, with no procedure-related complications [25]. However, the inability to maintain the initial response could be due to the ongoing disease process which affects the regenerated hepatocytes over a long period. Therefore, in order to maintain the effect of such therapies, it may be important to treat the causative liver disease, such as viral hepatitis, to make the infused cells resistant to viral infection [14] or to repeat the injection of stem cells at intervals to achieve sustained response [15] as previously reported in patients with large acute myocardial infarction [16].

We therefore sought to assess the effectiveness of HSC transplantation when given through two different routes in combination (peripherally and intra-portal vein) or sequentially (4 months apart) compared with the single-infusion procedure.

We found a statistically significant difference between the single-infusion and the repeated-infusion groups regarding serum albumin throughout the follow-up period. The maximum improvement in s-albumin occurred after 6 months of the study in G-I and then started to decline, whereas in G-II the improvement continued throughout the whole follow-up period to reach a maximum at the end of the study (after 1 year). This was also true regarding s-bilirubin, which revealed maximum improvement after 6 months in G-I (single infusion) followed by a decline, whereas in G-II (repeated infusion) the improvement continued throughout the follow-up period to reach the maximum at the end of the study. Similar results were previously reported by Levicar et al. [14] and Pai et al. [24].

Similarly, the maximum improvements in the INR and the MELD score occurred after 3 months of infusion in G-I (single infusion) and then started to decline, whereas in G-II (repeated infusion) the improvement continued throughout the follow-up period to reach the maximum at the end of the study. Our results in this context are in agreement with our previous studies by Salama et al. [18, 25, 26].

Our results regarding the maximum improvement in the Child–Pugh score which occurred after 6 months of follow-up are comparable with Terai et al. [6] and Pai et al. [24], who demonstrated significant improvement of Child–Pugh score at 4 and 24 weeks after autologous BMSC transplantation therapy.

All patients presenting with massive ascites (five patients) showed disappearance of their ascites after 2 months from starting therapy. By the end of the study, 40 % of the patients showed improvement in the degree of ascites, 60 % showed no change and none of the studied patients showed deterioration in their degree of ascites. Our findings in this context support previously published data [6, 24, 27].

During the follow-up period in the current study, 11 patients suffered attacks of haematemesis, which occurred after 6 months of the stem cell transplantation: four patients (13.3 %) from G-I, two patients (6.7 %) from G-II and five patients from the control group. Two patients were Child–Pugh score B and nine patients were Child–Pugh score C; two of which died from uncontrolled bleeding that occurred at the 12th month. Literature review showed that transient portal hypertension resulting from blood flow occlusion following stem cell infusion usually resolves in 2–3 hours [28]. At the beginning of the current study, there was no PVT in the studied groups. After 12 months of the study, the incidence of PVT was 3.3 % (one patient) in G-I compared with 6.7 % (two patients) in the control group. Further investigations for this patient by triphasic CT scan showed no hepatic focal lesions and an AFP level of 19 ng/ml. Patients in G-II (repeated infusion) showed no incidence of PVT throughout the follow-up period.

Although some studies in the literature have mentioned a possible carcinogenic effect of BMSC infusion in patients with chronic liver diseases, especially if in vitro pre-expanded [11], our data showed that after 12 months of follow-up by ultrasonography and s-AFP only one patient (3.3 %) in G-I (single infusion) and none in G-II (repeated infusion) were diagnosed with HCC compared with two patients (6.7 %) in the control group. Our results are comparable with those of Levicar et al. [14], who showed absence of any focal lesions in five patients treated by stem cell therapy who were followed-up by CT scan and serum AFP for 18 months, indicating that the stem cell product used was safe in the short term and over the long term, by absence of tumour formation. Our results suggest that combined application of HSCs by infusion into the portal vein and the peripheral veins bears the potential for augmenting liver regeneration in the clinical setting and can be used in the treatment of decompensated liver cirrhosis.

The HSCs can be delivered via different routes; each of which has its own advantages and disadvantages. The ideal strategy for stem cell delivery is that it should be easy to perform, minimally invasive, has very little side effects, has high cell survival and should be based on clinical settings. Moreover, under certain circumstances, the combination of more than one method may be considered [29, 30].

Two recently published meta-analyses have confirmed our previous and current data regarding the beneficial effect of stem cell therapy for end-stage liver disease patients and show almost the same results as ours. However, our current study is a step ahead of any previous published data by using a double stem cell infusion technique to extend the beneficial effect for more than 1 year [31, 32].

To our knowledge, the current study is the first trial investigating a second session of HSC infusion in Egyptian patients with HCV-related liver cell failure. Our results provide evidence for the first time that two infusions with HSC result in more sustained improvement in liver functions and quality of life during 12-months follow-up than a single infusion.

A point of criticism for the current study could be the lack of a control group of patients who received G-CSF alone, especially in the presence of several studies demonstrating a beneficial effect of G-CSF alone [33, 34]. One reason for not using G-CSF alone in the current study is that we have previously shown that the content of stem cells in end-stage liver disease patients is usually very low (1–3 % only). This amount is not sufficient for stem cell treatment in those patients and does not induce much improvement at the clinical and/or biochemical levels. However, with G-CSF the stem cell content increased to about 50–75 %, which provided the proper amount required for stem cell transplantation and differentiation even after purification of CD133^+^ and CD34^+^ cells. In addition, the main aim of the current study was to assess the beneficial effect (clinical and biochemical) of repeated MSC infusion, administered 4 months apart, as compared with single infusion and a control group.

## Conclusions

Repeated HSC infusions give more sustained clinical efficacy and improvement in liver functions and quality of life during 12-month follow-up compared with single HSC infusion. Accordingly, stem cell therapy could be a promising therapeutic modality in patients with end-stage liver disease, which may replace or decrease the need for whole organ transplantation in the future, especially if gene manipulation of the stem cells makes them able to resist viral infection or HCC development.

## Abbreviations

AFP: α-fetoprotein
ALT: Alanine transaminase
AST: Aspartate transaminase
BM: Bone marrow
BMSC: Bone marrow stem cell
CT: Computed tomography
G-CSF: Granulocyte colony-stimulating factor
HBcAb: Hepatitis B core antibody
HBsAg: Hepatitis B surface antigen
HCC: Hepatocellular carcinoma
HCV: Hepatitis C virus
HRS: Hepatorenal syndrome
HSC: Haematopoietic stem cell
INR: International Normalized Ratio
MELD: Model for End-Stage Liver Disease
MSC: Mesenchymal stem cell
PVT: portal vein thrombosis
SBP: Spontaneous bacterial peritonitis
WHO: World Health Organization
